# Locally advanced cholangiolocellular carcinoma successfully treated with curative resection after downsizing chemotherapy: a case report

**DOI:** 10.1186/s40792-021-01120-y

**Published:** 2021-01-26

**Authors:** Yuto Hozaka, Yota Kawasaki, Satoshi Iino, Tetsuya Idichi, Yuki Hirase, Kiyonori Tanoue, Yuko Mataki, Hiroshi Kurahara, Kosei Maemura, Takaaki Arigami, Shinichi Ueno, Shoji Natsugoe, Takao Ohtsuka

**Affiliations:** 1 Department of Digestive Surgery, Breast and Thyroid Surgery, Graduate School of Medical and Dental Sciences, Kagoshima University, 8-35-1, Sakuragaoka, Kagoshima, 890-8520 Japan; 2 Department of Clinical Oncology, Graduate School of Medical and Dental Sciences, Kagoshima University, Kagoshima, Japan

**Keywords:** Cholangiolocellular carcinoma, Cholangiocellular carcinoma, Hepatocellular carcinoma, Locally advanced, Downsizing chemotherapy, Gemcitabine, Cisplatin, S-1

## Abstract

**Background:**

Cholangiolocellular carcinoma (CoCC) is an extremely rare disease comprising less than 1% of all primary malignant liver tumors. No effective treatment other than resection has been established. Herein, we report a case of locally advanced CoCC diagnosed as unresectable, which was successfully treated with curative resection after downsizing chemotherapy.

**Case presentation:**

A 59-year-old Japanese woman with chronic hepatitis B was diagnosed with locally advanced intrahepatic cholangiocellular carcinoma. As it was difficult to perform R0 resection in the local hospital, chemotherapy combined with gemcitabine plus cisplatin was administered every 3 weeks. After a total of 10 courses of chemotherapy over 10 months the tumor was shown to be reduced in size by computed tomography imaging, and she was referred to our department for surgical resection. The effect of chemotherapy was classified as a “partial response” in the response evaluation criteria of solid tumors. After adding one course of chemotherapy, an extended left hepatectomy with resection of the caudate lobe was performed. R0 resection was achieved. Based on the pathological findings, the final diagnosis of CoCC was determined and eight courses of S-1 adjuvant chemotherapy were administered. At 14 months after the operation, the patient was alive without tumor recurrence.

**Conclusions:**

Downsizing chemotherapy with gemcitabine and cisplatin may be an effective treatment strategy in locally advanced CoCC. Further evidence is required to establish an optimal strategy for the treatment of locally advanced CoCC.

## Background

Cholangiolocellular carcinoma (CoCC) is speculated to originate from hepatic progenitor or stem cells [1, 2]. CoCC is an extremely rare disease which comprises less than 1% of primary malignant liver tumors, and surgical resection is the only curative treatment [1]. Compared to intrahepatic cholangiocellular carcinoma (ICC), patients with resectable CoCC tend to have a better prognosis [3]. On the other hand, there are limited reports of unresectable CoCC treatment, and no effective treatment has been established for advanced CoCC. Here, we report a rare case of locally advanced CoCC diagnosed as unresectable, which was successfully treated with hepatectomy after downsizing chemotherapy with gemcitabine plus cisplatin.

## Case presentation

**Fig. 1:**
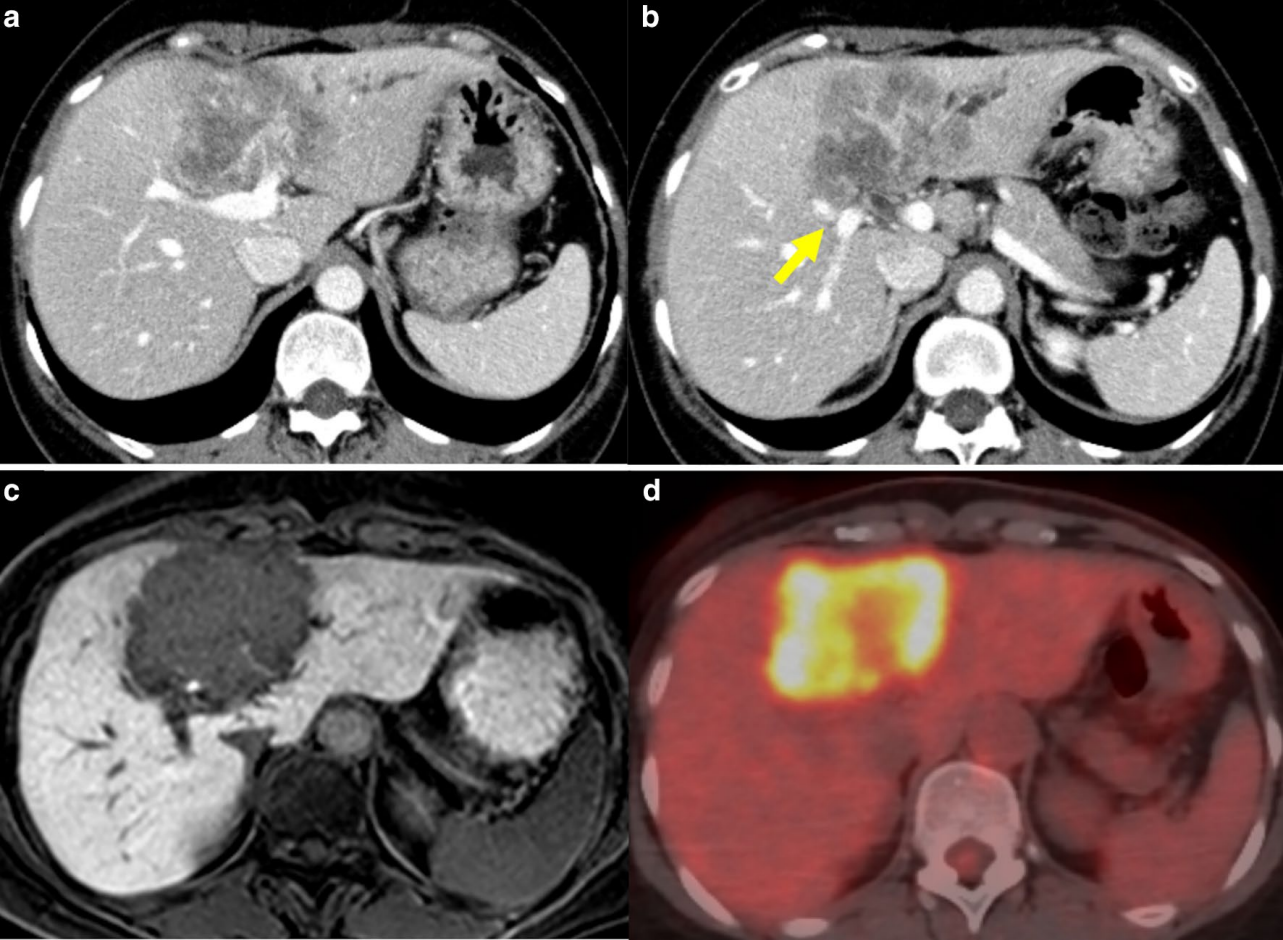
Images at admission of local hospital. **a** Contrast-enhanced abdominal computed tomography (CT) showed the tumor was mosaic enhancement, measured 65 × 61 mm in the hepatic segments 4 and 5 and 8. **b** Contrast-enhanced abdominal CT showed the tumor was attached to the biliary confluence, bifurcation of anterior–posterior Glissonian (yellow arrow) and right portal vein trunk. It was suspected to invasion to the left portal vein trunk, left intrahepatic bile duct and middle hepatic vein. **c** Magnetic resonance imaging (MRI) showed the tumor was hypointense on T1-weighted images. The margin of the tumor was unclear. **d** ^18^F-fluorodeoxyglucose (FDG)-positron emission tomography/computed tomography showed high FDG uptake of tumor with standardized uptake value-max of 11.15

A 59-year-old Japanese woman was admitted to a local hospital for evaluation of a liver mass detected during a regular medical examination. She had chronic hepatitis B and was treated with tenofovir. Abdominal enhanced computed tomography (CT) showed that the tumor had mosaic enhancement, with hepatic segments 4, 5, and 8 measuring 65 × 61 mm (Fig. 1a, b). The tumor was attached to the biliary confluence, bifurcation of the anterior–posterior Glissonian, and right portal vein trunk. An invasion to left portal vein trunk, left hepatic duct, and middle hepatic vein was suspected. The possibility of invasion into the right portal vein trunk could not be ruled out. No obvious invasion into the proper and right hepatic arteries was observed. Results showed that the tumor invaded the right anterior hepatic duct and right posterior hepatic duct (RPHD). Magnetic resonance imaging (MRI) showed that the tumor was hypointense on T1-weighted images and hyperintense on T2-weighted images (Fig. 1c). The margin between the tumor and the normal hepatocyte was unclear. ^18^F-fluorodeoxyglucose (FDG)-positron emission tomography/computed tomography (PET/CT) showed high FDG uptake with a standardized uptake value (SUV) of 11.15 (Fig. 1d). A liver biopsy of the tumor revealed moderate to poorly differentiated adenocarcinoma that showed a tubular pattern that comprised small variably sized, irregular tubules in the fibrous stroma. On the basis of the above findings, the patient was diagnosed with mass-forming type ICC, T4N0M0 stage IIIB according to the 8th edition of the Union for International Cancer Control (UICC). At the time of diagnosis, indocyanine green (ICG) angiography for evaluating the clearance rate of ICG and technetium-99m-galactosyl human serum albumin (Tc-99m GSA) scintigraphy for evaluating the liver reserve were not performed; however, according to CT volumetry, the volume of the entire liver was 839 mL, while the volume of the posterior segment was only 200 mL. Curative resection by left trisectionectomy was anatomically possible; however, if performed, the remnant liver volume and the future liver remnant were predicted to be only 200 mL and 23.8%, respectively. Thus, it was difficult to perform an R0 resection in this patient. Chemotherapy combined with gemcitabine (GEM 1000 mg/m^2^, days 1 and 8) plus cisplatin (CDDP 25 mg/m^2^, days 1 and 8) (GC) was administered every 3 weeks. There were no serious adverse events during the course of chemotherapy. CT was performed once every 3–4 courses, and it was confirmed that the tumor was gradually reducing in size.

After a total of 10 courses of combination chemotherapy over 10 months, she was referred to our department for resection. Physical examination upon admission was normal. Hepatitis C virus antibodies were negative. Hepatitis B virus surface antigen and core antibody were positive. The serum tumor markers at the time of admission to our hospital were normal as follows: prothrombin induced by vitamin K absence or antagonist-II, 16 mAU/mL; α-fetoprotein, 2.6 ng/mL; carbohydrate antigen 19–9, 13.0 U/mL. Other laboratory results were normal. Her indocyanine green retention rate at 15 min (ICGR15) was 5.0%. According to the Child–Pugh classification, her liver function was stage A. She had grade A liver damage. Tc-99m GSA scintigraphy revealed the following results for the clearance index (HH15) and the receptor index (LHL5)—HH15: 0.544 (normal level > 0.5) and LHL15: 0.931 (normal level > 0.9). Thus, it was concluded that the liver reserve was preserved.

**Fig. 2:**
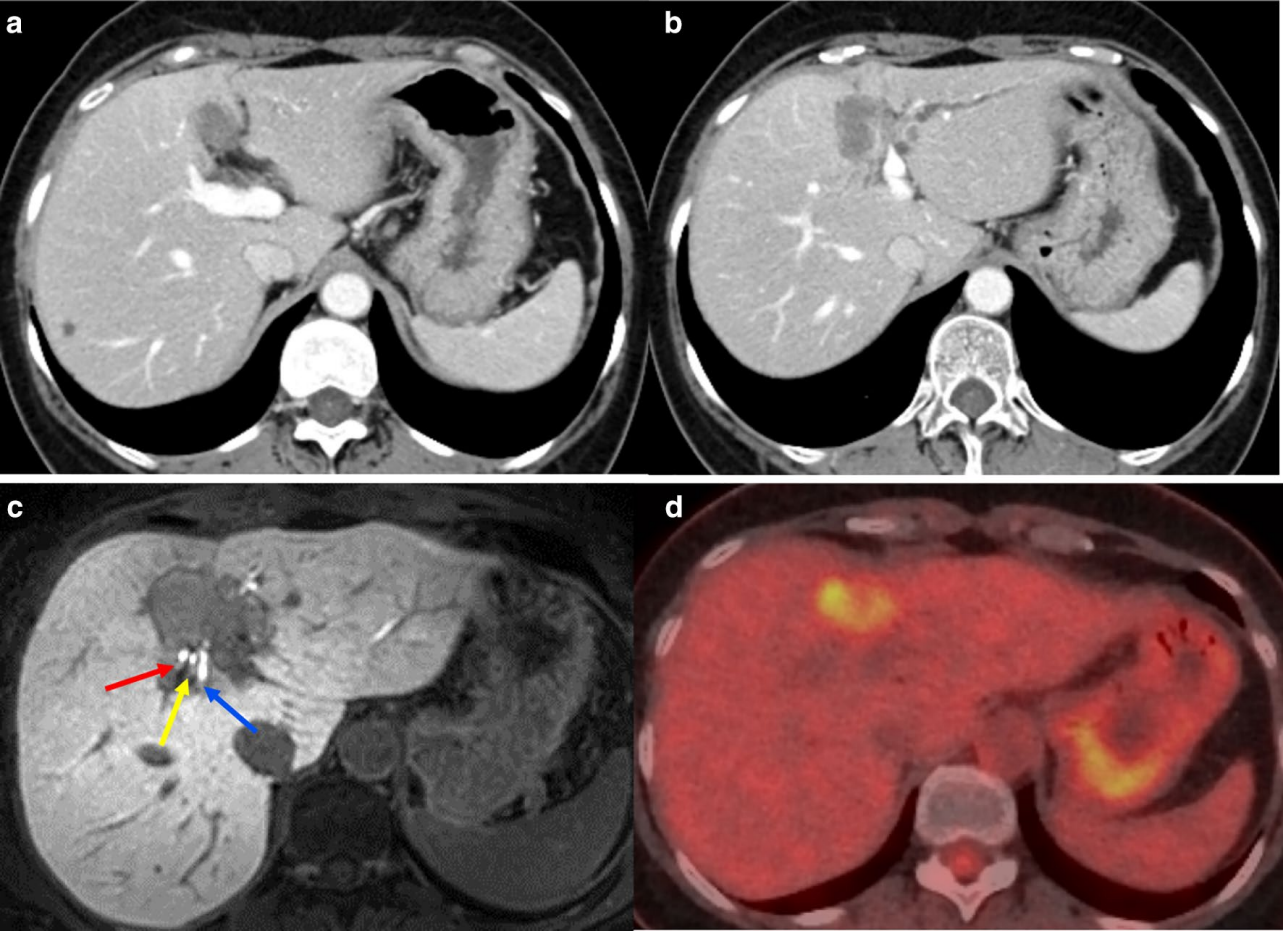
Images after total 10 courses of the combination chemotherapy with gemcitabine plus cisplatin. **a**, **b** Contrast-enhanced abdominal CT showed the tumor had decreased to 38 × 35 mm (42% reduction). **c** The tumor was close to the hilar plate. But no findings of invasion to right anterior dorsal bile duct (red arrow), right anterior ventral bile duct (yellow arrow) and right posterior hepatic duct (blue arrow) were observed magnetic resonance imaging. **d** FDG-PET/CT showed FDG uptake of tumor had decreased SUV max 6.5

**Fig. 3:**
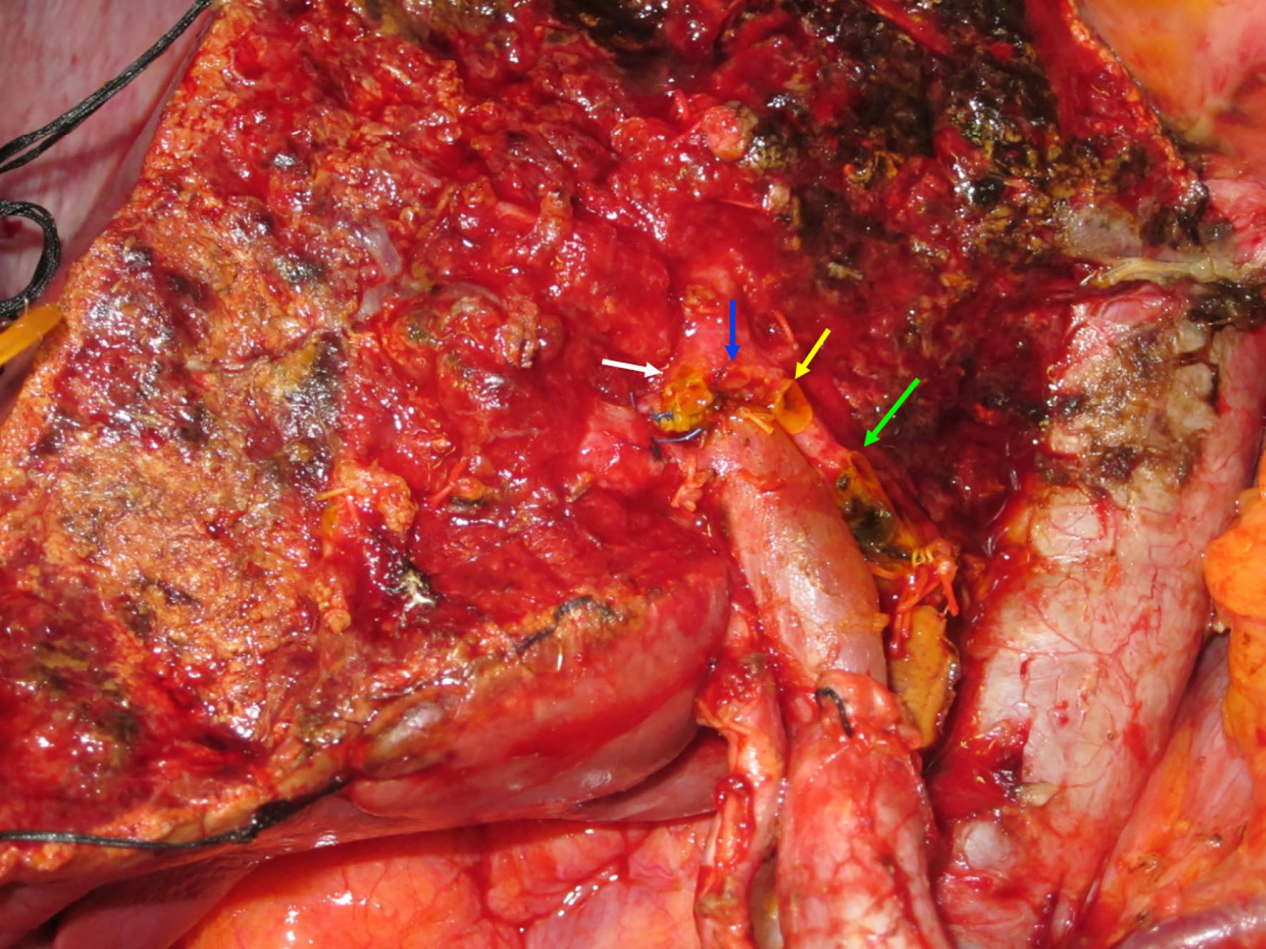
Operation findings. This image shows the operative findings after extended left hepatectomy with resection of the caudate lobe, resection of the extrahepatic bile duct, and local-regional lymphadenectomy of the hepatoduodenal ligament. B5 (white arrow), B8a (blue arrow), B8c (yellow arrow), and right posterior bile duct (green arrow), were reconstructed by bilioenteric anastomosis

The size of the primary tumor had decreased to 38 × 35 mm (42% reduction). The tumor was close to the hilar plate and bifurcation of the anterior–posterior Glissonian, but farther than before chemotherapy on CT and MRI images (Fig. 2a–c). We considered that the tumor was unlikely to infiltrate the B8 and B5 levels of bile ducts and RPHD. FDG-PET/CT revealed that FDG uptake of the tumor had decreased (SUV max: 6.5) (Fig. 2d). Although the diagnosis after chemotherapy did not change the T4N0M0 stage IIIB (according to UICC), the effect of chemotherapy was classified as a “partial response” in the response evaluation criteria in solid tumors (RECIST). On the basis of the above findings, if extended left hepatectomy with resection of the caudate lobe could be performed (because we believed that the tumor was unlikely to infiltrate the B8 and B5 levels of the bile ducts and RPHD), we determined that a radical resection was possible. If extended left hepatectomy with resection of the caudate lobe was performed after chemotherapy, the entire liver volume (as measured by CT volumetry), remnant liver volume, and future liver remnant were predicted to be 857 mL, 436 mL, and 50.8%, respectively. To predict the remnant liver function, our facility uses the KGSA value (i.e., the ICG clearance rate using galactosyl), and the remnant KGSA (KGSA value × functional rate of the remnant liver), as reported by Okabayashi et al. [4]. If the remnant KGSA is 0.05 or more, it is used as an index for favorable post-hepatectomy liver function. In this case, the remnant KGSA was 0.079, and it was determined that an extended left hepatectomy with caudate lobe resection was possible. After adding one course of chemotherapy, extended left hepatectomy with resection of the caudate lobe, resection of the extrahepatic bile duct, and local-regional lymphadenectomy of the hepatoduodenal ligament were performed (Fig. 3). Biliary reconstruction was performed with bilioenteric anastomosis in the B5, B8, and RPHD.

**Fig. 4:**
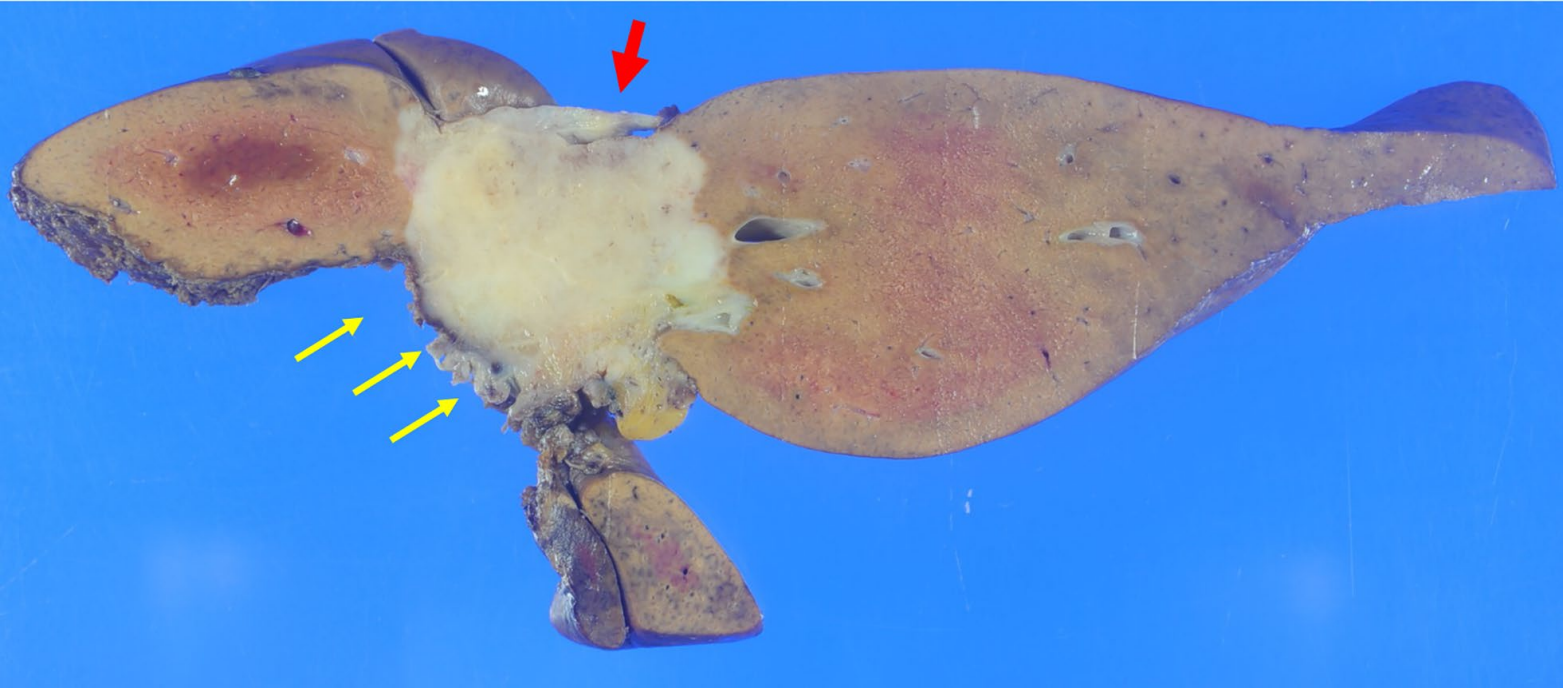
Gross findings. The resected specimens measured 4.5 × 3.5 cm in tumor size. The tumor was a whitish, solid and mass-forming (red arrow). There was no tumor exposure on the transected plane (yellow arrows)

**Fig. 5:**
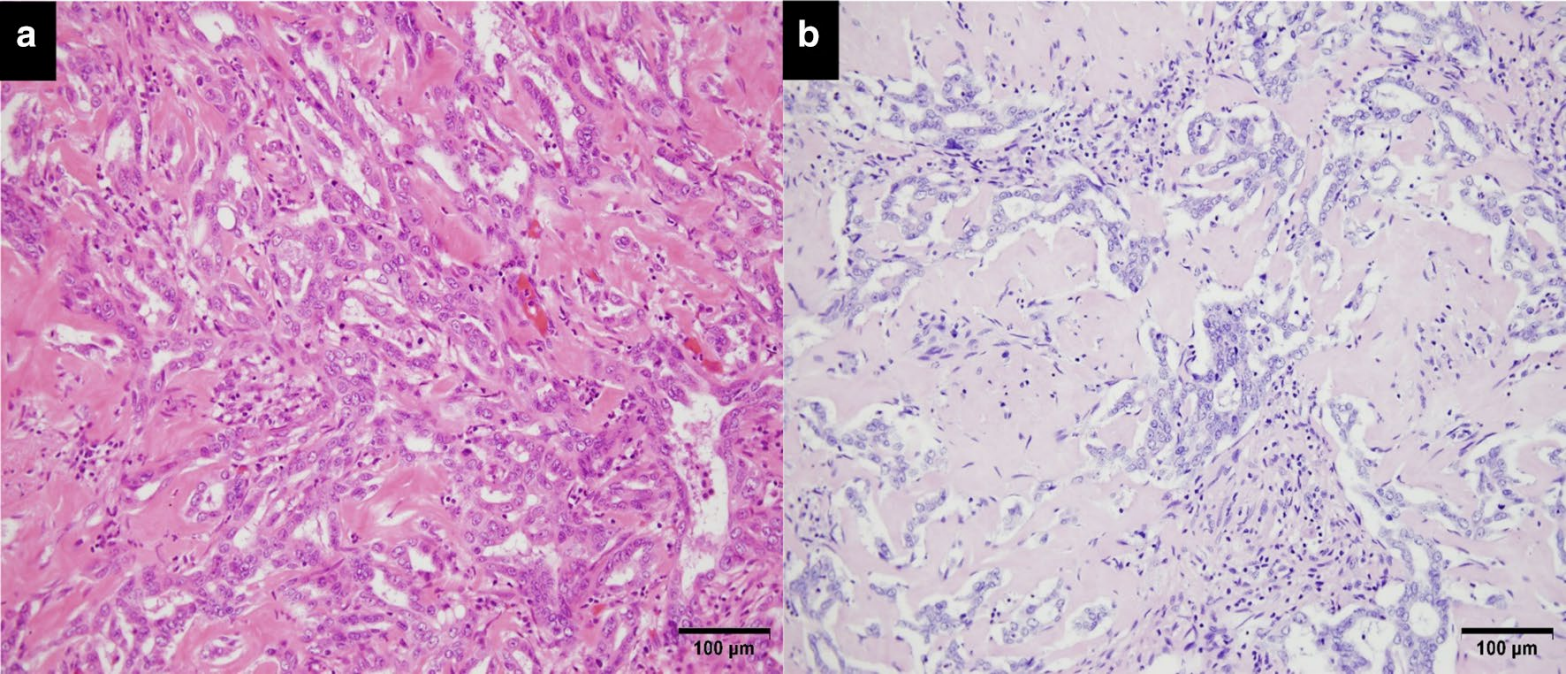
Histopathological findings. **a** Microscopic findings (HE staining ×100) showed proliferation of the tumor cells, composed of irregular small tubules with an anastomosing and a cord-like pattern, indicating a moderately to poorly differentiated adenocarcinoma. **b** Microscopic findings (mucicarmine staining ×100) showed mucin production was not observed

The surgical specimens showed a whitish solid tumor of the mass-forming type, measuring 4.5 × 3.5 cm in size (Fig. 4). Pathological examination of the specimens revealed a hyalinized scar tissue with necrosis that comprised most part of the tumor, and proliferation of the tumor cells in a small tubular or cord-like pattern (with a slit-like lumen) peripherally around the scar. Small tubules were composed of cuboidal tumor cells, which have round nuclei and scant cytoplasm. An irregular anastomosing pattern was observed frequently. Although the tumor was predominantly replaced by the hyalinized scar (which probably reflected the effect of chemotherapy), the tumor cells that had proliferated peripherally around the scar were composed of a small ductular component intermixed with a cord-like structure. A large duct-type component with mucin-secreting glands, which is typically seen in ICC, was not observed in the current case (Fig. 5a, b). Immunohistochemically, the tumor cells were positive for EMA and CD56 (NCAM). Based on the above findings, the tumor was diagnosed as a CoCC. Approximately one-third of the tumor was composed of viable tumor cells. The surgical margin was 1 mm, and R0 resection was achieved. Lymph nodes showed no metastasis.

Although mild bile leakage was observed during the postoperative course, bile leakage was discontinued after conservative treatment, and the patient was discharged on the 44th postoperative day. Adjuvant chemotherapy performed eight courses (4-week administration and 2-week withdrawal) of S-1 (TS-1; tegafur, gimeracil, oteracil potassium), at 100 mg/body per day. Approximately 14 months after the operation, abdominal CT and PET/CT showed no signs of tumor recurrence.

## Discussion

CoCC was first reported by Steiner and Higginson in 1959 and is more likely to occur in cases of chronic hepatitis [1, 2]. There are a few reports that show chemotherapy is effective for advanced CoCC; however, there is no established choice of chemotherapy [5, 6]. Therefore, focusing on the current case, we discuss three points in CoCC: radiographic imaging features, treatment including chemotherapy, and prognosis.

First, there are several reports on the diverse imaging findings of CoCC [7–10]. CoCC is speculated to originate from hepatic progenitor or stem cells, which have stem cell features and can differentiate into both hepatocytes and cholangiocytes [1, 11]. Thus, CoCC often proliferates heterogeneously and has a contained hepatocellular (HCC)-like area and ICC-like area in a part of the nodules without mucus, respectively. Therefore, CoCCs can show dual characteristics of HCCs and ICCs in images, such as whole early enhancement with delayed washout and peripheral early enhancement with centripetal filling, respectively [1]. These findings are considered to depend on cellularity and the amount of fibrous stroma. Because the image findings for CoCC are often similar to those for ICC or HCC, it is often difficult to preoperatively diagnose CoCC [7]. On the other hand, it has been reported that some image findings are characteristic of CoCC. Kozaka et al. defined “pure CoCC” as a tumor that consists exclusively of CoCC without any HCC or ICC components [8]. The characteristics of pure CoCC in CT findings that were revealed and compared with ICC were hypervascularity, peritumoral enhancement in the arterial phase, presence of intra-tumoral portal tracts, rare intrahepatic bile duct dilatation, and prolonged staining in the late phase. Additionally, MRI shows isointensity or hypointensity on T1-weighted images and hyperintensity on T2-weighted images [12]. In the current case, CT showed that the tumor had a mosaic enhancement pattern rather than a peritumoral enhancement pattern. MRI showed that the tumor was hypointense on T1-weighted images and hyperintense on T2-weighted images. The findings of MRI were pure CoCC findings, but the findings of CT were not typical pure CoCC findings; thus, it was difficult to distinguish CoCC preoperatively. If cases with chronic hepatitis do not have the typical imaging findings of HCCs or ICCs, CoCC should be listed as a differential diagnosis to confirm these characteristic findings.

Second, regarding treatment, curative surgery is the first choice for patients with CoCC. There is no evidence of other treatments, and reports of unresectable CoCC are limited. We searched the PubMed database for published literature using the terms “cholangiolocellular carcinoma” and “chemotherapy” between 1950 and October 2020. To the best of our knowledge, there have been three reports of advanced CoCC in which radical surgery was performed after chemotherapy, including the current case (Table 1) [6, 13]. With the exception of the current case, systemic chemotherapy with gemcitabine alone was administered in one patient, and chemotherapy combined with gemcitabine plus cisplatin via hepatic arterial infusion was administered in one patient. In one case of hepatic arterial infusion therapy, the tumor size was almost unchanged before and after chemotherapy. Another case with gemcitabine monotherapy did not describe the tumor size after chemotherapy. The current case was initially diagnosed with unresectable ICC, and GC was administered, according to the ABC-02 trial. The ABC-02 trial has shown that compared to gemcitabine alone, GC significantly improved overall survival for advanced biliary tract cancer (BTC) including ICC in 2010 [14]. In addition, there is a report of a group of cases in which conversion surgery could be performed for unresectable BTC after the use of GC [15]. As mentioned above, GC is an effective treatment, but there are some cases in which the effect is poor. The 2018 October European Society for Medical Oncology (ESMO) reported the results of a randomized phase III comparative study on gemcitabine, cisplatin plus S-1 (GCS) versus GC for advanced BTC. The median overall survival of the GCS arm was significantly longer than that of the GC arm, indicating a better prognosis in the former. Additionally, the percentage of partial responses in the 123 patients enrolled in the GC arm was 15.0%, while the percentage of partial responses in the 119 patients enrolled in the GCS arm was 41.5%; thus, GCS was concluded to achieve a better disease control as compared to GC [16]. Therefore, GCS is used as one of the standard treatments for advanced BTC instead of GC. In the current case, because there was no history of triplet cytotoxic chemotherapeutic regimens like GCS at the local hospital at the time of diagnosis, GC was administered; however, GCS may have been a better treatment option, considering that the incidence of adverse events, except those related to S-1, did not differ significantly between the two arms of the phase III study. In the current case, CoCC showed many elements similar to ICC clinically, and required histopathological and immunohistochemical assessments as well as a special staining for mucin to differentiate it from ICC. When there are many ICC-like areas, the effect of combination therapy of gemcitabine and cisplatin can be expected to have efficiency similar to that in ICC. In addition, because CoCC is associated with the expression of cholangiocyte markers, such as CK7 and CK19, chemotherapy regimens generally used for ICC may be effective for CoCC [17].

**Table 1:** Summary of clinical features of cases with CoCC who underwent surgery after chemotherapy

No.	Author [reference number], year	Age	Sex	Viral infection	Underlying liver disease	Localization of tumor	Size (cm) of tumor before chemotherapy	Size (cm) of tumor after chemotherapy	Purpose of chemotherapy	Administration method/regimen/total courses	Surgical treatment	Adjuvant chemotherapy	Outcome
1	Nakayama [6], 2012	78	M	HCV	nd	S4/S5	8.0×	7.0×	Downsizing chemotherapy	Hepatic arterial infusion/gemcitabine and cisplatin/3 courses	Central bisegmentectomy	None	Being followed up for 3 months
2	Kawashima et al. [13], 2012	57	F	nd	nd	S1/S5/S6	7.0×	nd	Neoadjuvant chemotherapy	Intravenous/gemcitabine/ 2 courses	Right hepatectomy	Gemcitabine	Being followed up for 4 months
3	Current case	59	F	HBV	Chronic hepatitis	S4/S5/S8	6.5	3.8	Downsizing chemotherapy	Intravenous/gemcitabine and cisplatin/11 courses	Extended left hepatectomy	S-1	Being followed up for 14 months

*nd* not described, *HBV *Hepatitis B virus, *HCV* Hepatitis C virus, *CoCC* cholangiolocellular carcinoma

Third, the overall prognosis for CoCC has not yet been clearly determined due to its low incidence, but several reviews have reported that the prognosis is comparatively better than that of ICC [3, 18, 19]. Ariizumi et al. reported that patients with CoCC showed favorable long-term survival after curative surgery; 28 cases of CoCC had a 5-year survival rate of 75% [3]. Komuta et al. reported that one of the independent risk factors for recurrence was a maximal tumor size of > 40 mm [1]. Although not limited to CoCC, including ICC, Chen et al. and Kusano et al. reported microvascular invasion as a risk factor for recurrence [18, 19]. In the current case, the diameter of the tumor was 45 mm after chemotherapy; microvascular invasion was also present; thus, we would expect the current case to have a high risk of recurrence. Although the effectiveness of adjuvant therapy for BTC has been shown, no standard adjuvant chemotherapy regimen has been established for patients with BTC or CoCC [20]. Additionally, although clinical trials (JCOG1202) are currently in progress and no results have been obtained, S-1 is considered to be a promising agent for postoperative adjuvant therapy in patients with BTC [21]. In the current case, considering the risk of recurrence, according to JCOG1202, S-1 as adjuvant chemotherapy was administered.

## Conclusions

We report a rare case of locally advanced CoCC that had been diagnosed as unresectable, which was successfully treated with hepatectomy after downsizing chemotherapy. Downsizing chemotherapy with gemcitabine and cisplatin may be an effective treatment strategy for locally advanced CoCC. Further evidence is required to establish an optimal strategy for the treatment of locally advanced CoCC.

## Data Availability

The datasets supporting the conclusions of this article are included within the article and its additional files.

## Abbreviations

CoCC: Cholangiolocellular carcinoma
ICC: Intrahepatic cholangiocellular carcinoma
CT: Computed tomography
GC: Gemcitabine, cisplatin
RPHD: Right posterior hepatic duct
MRI: Magnetic resonance imaging
FDG18: ^18^F-fluorodeoxyglucose
PET/CT: Positron emission tomography/computed tomography
SUV: Standardized uptake value
UICC: Union for International Cancer Control
ICG: Indocyanine green
Tc-99m GSA: Technetium-99m-galactosyl human serum albumin
HCC: Hepatocellular carcinoma
BTC: Biliary tract cancer
ESMO: European Society for Medical Oncology
GCS: Gemcitabine, cisplatin plus S-1
